# Supplementary material for: Involution of brown adipose tissue through a Syntaxin 4 dependent pyroptosis pathway
Source: Nat Commun. 2024 Apr 2;15:2856. doi: 10.1038/s41467-024-46944-y (PMC10987578; doi:10.1038/s41467-024-46944-y)
Supplement: Supplementary file 1 — Supplementary Information [file 41467_2024_46944_MOESM1_ESM.pdf]

Supplemental figures and figure legends

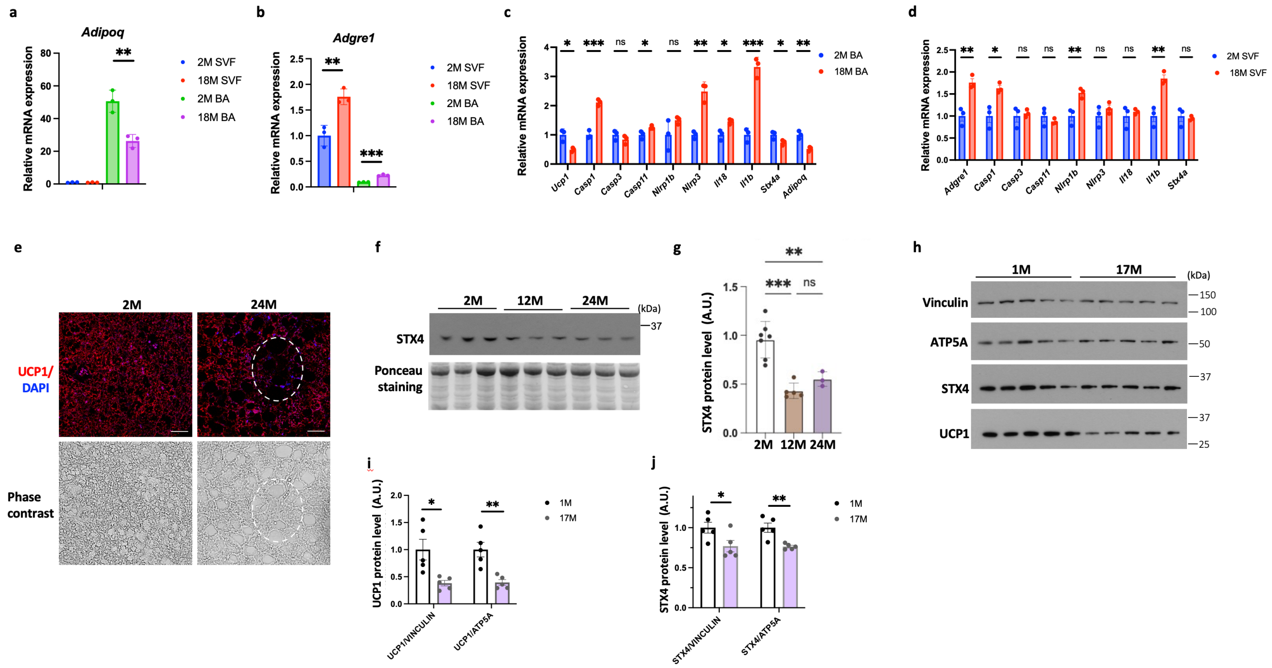

Supplemental Figure 1. Increased Caspase 1/11-mediated pyroptosis in both brown adipocytes and stromal vascular fraction of aging mice. a *Adipoq* mRNA and **b** *Adgre1* mRNA level of stromal vascular fraction (SVF) and brown adipocytes (BA) from 2- and 18- month-old wildtype male mice. n = 3 mice. **c** Purified brown adipocytes from WT male mice at 2 months and 18 months of age were extracted and subjected to qRT-PCR to determine the indicated mRNA levels. n = 3 mice. **d** Purified stromal vascular fraction from WT male mice at 2 months and 18 months of age were extracted and subjected to RT-qPCR to determine the indicated mRNA levels. n = 3 mice. **e** UCP1 immunofluorescence (red) and DAPI (blue) staining of nuclei in brown adipose tissue of WT male mice at 2 months and 24 months of age. Circles indicate perilipin-depleted cells. Scale bars: 100μM. **f** Immunoblots of STX4 protein of brown adipose tissue from 2-, 12-, and 24-months-old male mice. Ponceau's staining as loading control. **g** Quantitation of STX4 expression in brown adipose tissue at 2-, 12-, and 24-months-old male mice. n = 7 (2M), n = 5 (12M), n = 3 (24M) mice. **h** Immunoblot of STX4, UCP1, ATP5A and Vinculin in brown adipose tissue of WT female mice at 1 and 17 months of age. **i** Quantitation of UCP1 expression in brown adipose tissue at 1- and 17-month-old female mice. n = 5 mice. **j** Quantitation of STX4 expression in brown adipose tissue at 1- and 17-month-old female mice. n = 5 mice. All data represent the mean ± SEM. **P* < 0.05, ***P* < 0.01 and ****P* < 0.001, by two-tailed Student's *t* test (a, b, c, d, i, j) or one-way ANOVA (g).

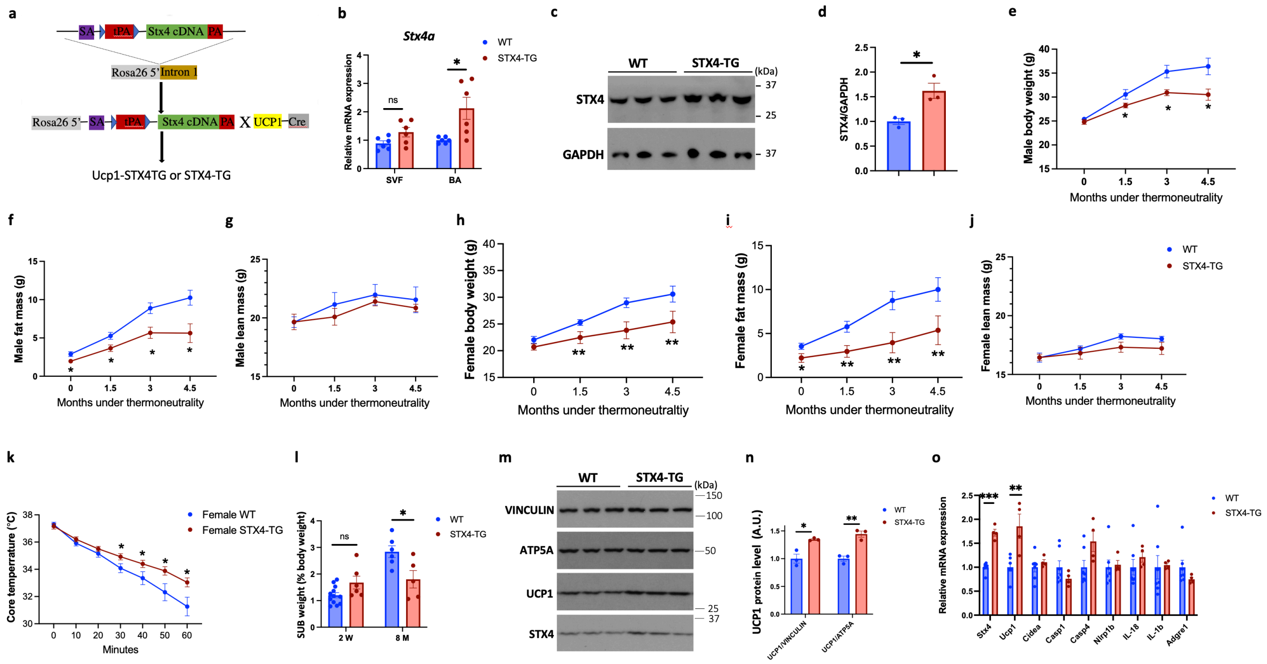

Supplemental Figure 2. Overexpression of STX4 in brown adipocytes preserve the function of brown adipose tissue under thermoneutrality and aging. a Schematic diagram of generating brown adipocyte specific Stx4a transgenic mice. **b** *Stx4a* mRNA in purified brown adipocytes (BA) and stromal vascular fraction (SVF) from BAT of 2-month-old WT and STX4-TG male mice. n = 6 mice. **c** Immunoblot of STX4 from BAT of 2-month-old WT and STX4-TG male mice. **d** Quantitation of STX4 protein levels in BAT of WT and STX4-TG male mice. n = 3 mice. **e** Body weight of WT and STX4-TG male mice maintained at thermoneutrality for up to 4.5 months. n = 5 mice. **f** Fat mass determined by Eco-MRI in male mice maintained at thermoneutrality for various time. n = 5 mice. **g** Lean mass determined by Eco-MRI in male mice maintained at thermoneutrality. n = 5 mice. **h** Body weight of WT and STX4-TG female mice under thermoneutrality. n = 6 (WT), n = 5 (STX4-TG) mice. **i** Fat mass determined by Eco-MRI in female mice maintained at thermoneutrality. n = 6 (WT), n = 5 (STX4-TG) mice. **j** Lean mass determined by Eco-MRI in female mice maintained at thermoneutrality. n = 6 (WT), n = 5 (STX4-TG) mice. **k** Core body temperature of female STX4-TG and WT mice subjected to acute cold exposures (room temperature to 0°C) for 60 minutes. n = 6 (WT), n = 5 (STX4-TG) mice. **l** Subcutaneous adipose tissue mass normalized to body weight in WT and STX4-TG female mice maintained at thermoneutrality for 2 weeks and 8 months. n = 12 (WT), n = 6 (STX4-TG) mice. **m** Immunoblot of STX4, UCP1, ATP5A and Vinculin in brown adipose tissue from WT and STX4-TG female mice at 17 months of age. **n** Quantitation of UCP1 expression in brown adipose tissue of WT and STX4-TG female mice at 17 months of age. n = 3 mice. **o** Brown adipose tissue from WT and STX4-TG female mice at 17 months of age were extracted and subjected to RT-qPCR to determine the indicated mRNA levels. n = 7 (WT), n = 4 (STX4-TG) mice. All data represent the mean ± SEM. *$P < 0.05$, **$P < 0.01$ and ***$P < 0.001$, by two-tailed Student's *t* test.

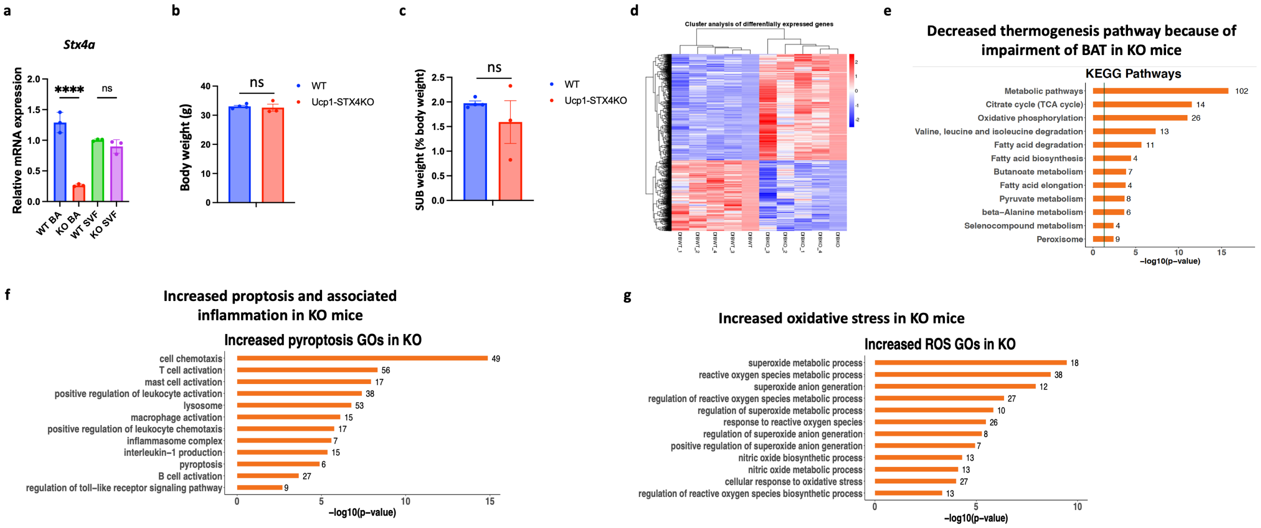

Supplemental Figure 3. Changed pathways in BAT of Ucp1-STX4KO (KO) mice compared to WT littermates by RNA sequencing. a mRNA level of *Stx4a* in purified brown adipocytes (BA) and stromal vascular fraction (SVF) from 2-month-old male WT and Ucp1-STX4KO mice. n = 3 biologically independent samples. **b** Body weight of WT and Ucp1-Stx4KO male mice at 5 months of age. n = 4 mice. **c** Subcutaneous fat mass normalized to body weight in WT and Ucp1-Stx4KO male mice at 5 months of age. n = 4 (WT), n = 3 (Ucp1-STX4KO) mice. **d** Heatmap of RNAseq data from BAT of 2-month-old male WT and Ucp1-STX4KO mice. n = 4 mice. **e** Decreased KEGG pathways in BAT of Ucp1-STX4KO mice compared to WT littermates. n = 4 mice. **f** Increased pyroptosis and inflammation pathways in BAT of Ucp1- STX4KO mice compared to WT littermates. n = 4 mice. **g** Increased oxidative stress pathways in BAT of Ucp1- STX4KO mice compared to WT littermates. n = 4 mice. All data represent the mean ± SEM. *$P < 0.05$, **$P < 0.01$ and ***$P < 0.001$, by two-tailed Student's *t* test.

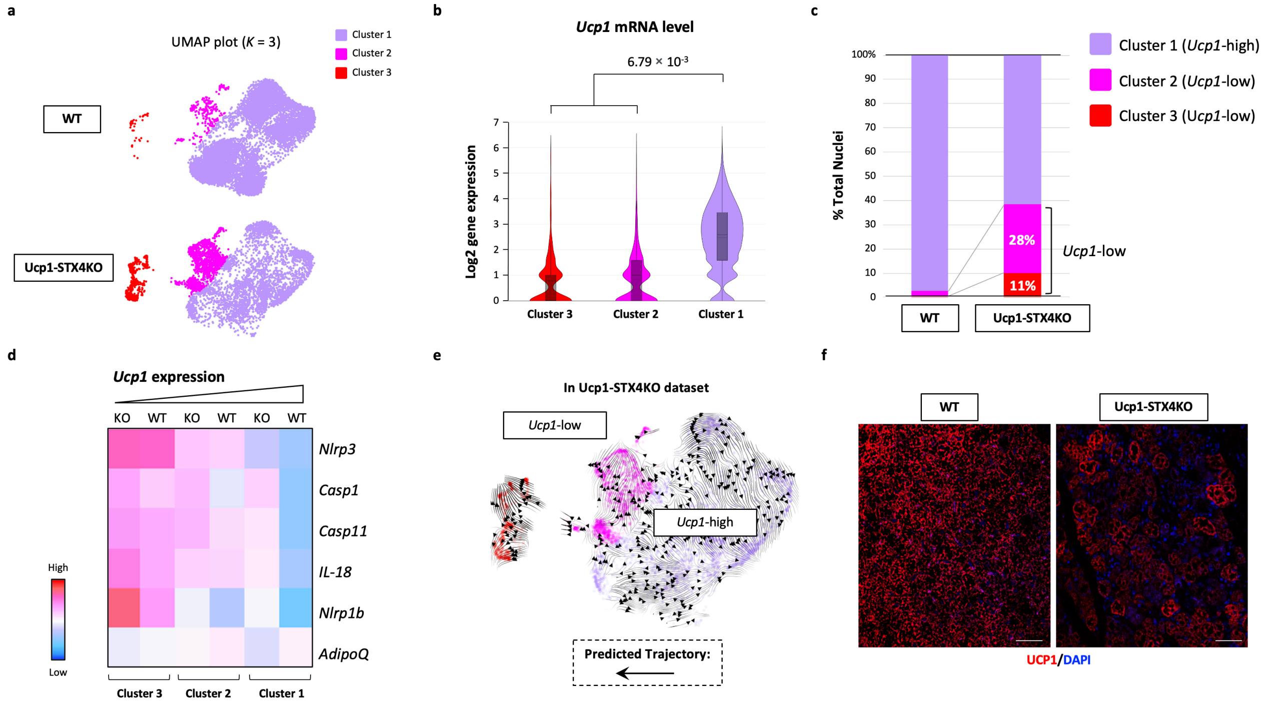

Supplemental Figure 4 Genetic ablation of STX4 in iBAT results in the presence of pyroptotic Ucp1-low brown adipocytes. a Uniform manifold approximation and projection (UMAP) projection of all 15,712 sequenced brown adipocyte nuclei split by genotype from 3-month-old iBAT from control (WT) and Ucp1-STX4KO (KO) male mice. **b** *Ucp1* mRNA level in different clusters. **c** The relative percentage of the adipocyte cells obtained in Panel A. **d** Heat map showing the expression of pyroptotic gene markers in the *Ucp1*-low and *Ucp1*-high expressing brown adipocytes. **e** Lineage relationships between cellular subtypes predicted by the RNA velocity algorithm. Arrows represent inferred cell interconversions. **f** Representative immunofluorescence image of iBAT from control (WT) and Ucp1-STX4KO male mice at 2 months of age. Scale bars: 100μM.

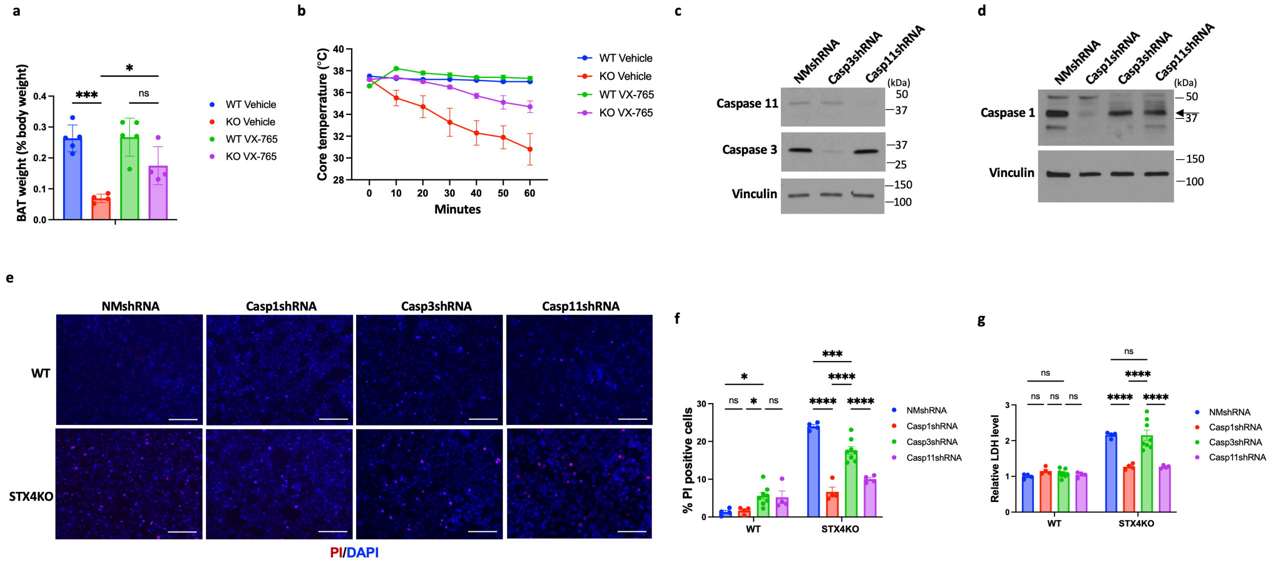

Supplemental Figure 5. CASP1/11 inhibition prevented the involution of brown fat mass in Ucp1-STX4KO mice. WT and Ucp1-STX4KO male mice at 1.5-month-old were injected with vehicle or VX-765 (50mg per kg of their body weight, three times a week) for 2 months. **a** Brown fat mass normalized to body weight of WT and Ucp1-STX4KO male mice treated with vehicle or VX-765. n = 5 (WT Vehicle and WT VX-765), n = 4 (KO Vehicle and KO VX-765) mice. **b** Core body temperature of WT and Ucp1-STX4KO male mice subjected to acute cold exposures (room temperature to 4℃) for 60 minutes. n = 4 mice. **c, d** Immunoblot for Caspase 11, Caspase 3, Caspase 1, and Vinculin in STX4fl/fl cells that have been transfected with NMshRNA, Casp1shRNA, Casp3shRNA or Casp11shRNA lentivirus. **e** The representative image of PI/Hoechst staining of STX4KO adipocytes being transfected with NMshRNA, Casp1shRNA, Casp3shRNA or Casp11shRNA lentivirus, at day 12 of differentiation. **f** Quantitation of the percentage of PI positive cells in panel E. About 500 cells from each group are counted. **g** The released LDH level from medium of STX4KO adipocytes being transfected with NMshRNA, Casp1shRNA, Casp3shRNA or Casp11shRNA lentivirus at day 12 of differentiation. n = 3 biologically independent samples. All data represent the mean ± SEM. *$P < 0.05$, **$P < 0.01$, ***$P < 0.001$ and ****$P < 0.001$, by one-way ANOVA (a) or two-way ANOVA (b, f, g).

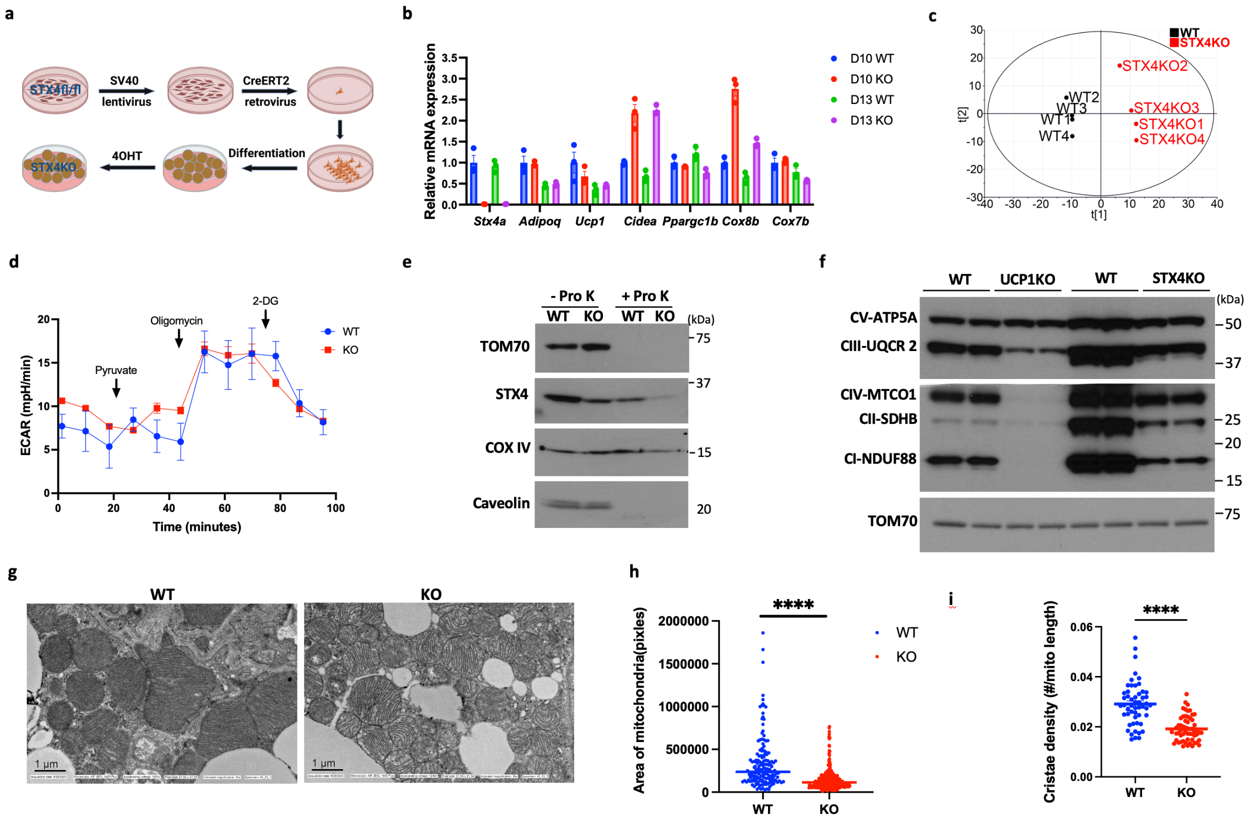

Supplemental Figure 6. STX4 can localize to the inner mitochondria membrane and regulates UCP1 protein stability.

a Schematic diagram of making immortal STX4KO brown adipocyte cell line. **b** Immortalized brown adipocytes from WT and STX4KO male mice were differentiated for 10 and 13 days, then RNA was extracted and subjected to qRT-PCR to determine the indicated mRNA levels. n = 3 biologically independent samples. **c** PLS-DA score of metabolites from brown adipocytes of WT and STX4KO male mice at day 12. n = 4. **d** Extracellular acidification rate (ECAR) determined by Seahorse XF Flux Analyzer from WT and STX4KO brown adipocytes using pyruvate as substrate. n = 4 (WT), n = 5 (STX4KO) biologically independent samples. **e** Isolated mitochondria from immortalized WT and STX4KO brown adipocytes differentiated for 12 days were treated with and without proteinase K 20mg/ml for 10 min and immunoblotted for the indicated proteins. **f** BAT mitochondria were isolated from 6-week-old WT, UCP1KO and Ucp1-STX4KO mice. The two sets of WT samples were the littermate controls for each genotype. The isolated mitochondria were then immunoblotted for several oxidative phosphorylation protein subunits and TOM70 as loading control as indicated. **g** Representative TEM of brown adipose tissue from WT and Ucp1-Stx4KO male mice at 3 months of age. **h** Quantification of mitochondria area of brown adipose tissue from WT and Ucp1-Stx4KO male mice at 3 months of age. (n = 160 (WT), n = 320 (Ucp1-Stx4KO) mitochondria). **i** Quantitative of cristae density as the number of cristae normalized to the length of mitochondria (pixels) of brown adipose tissue from male WT and Ucp1-Stx4KO mice at 3 months of age. All data represent the mean ± SEM. ****P < 0.0001 by two-tailed student's t test.

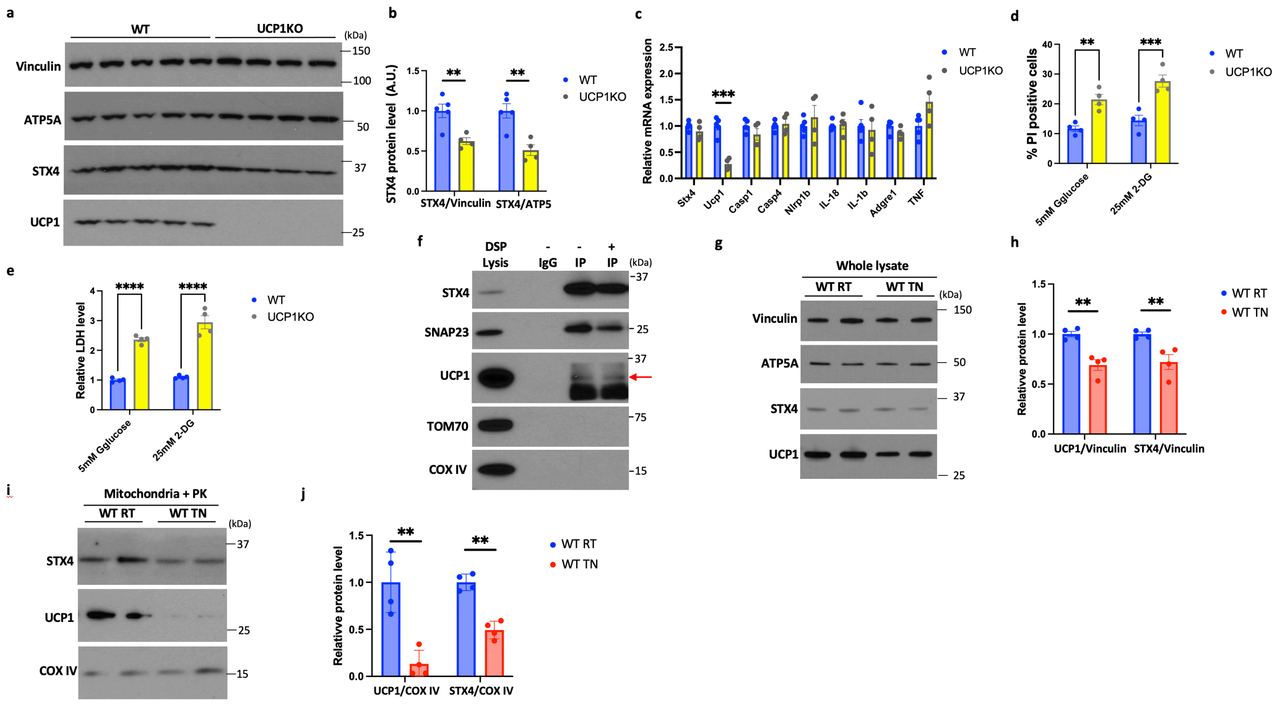

Supplemental Figure7. Thermoneutrality reduces both UCP1 and STX4 protein levels. a Immunoblot of STX4, UCP1, ATP5A and Vinculin in brown adipose tissue from WT and UCP1KO male mice at 2 months of age. **b** Quantitation of STX4 protein levels in brown adipose tissue of WT and UCP1KO mice at 2 months of age. n = 5 (WT), n = 4 (UCP1KO) mice. **c** Brown adipose tissue from WT and UCP1KO male mice were extracted and subjected to RT-qPCR to determine the indicated mRNA levels. n = 5 (WT), n = 4 (UCP1KO) mice. **d** Quantification for the percentage of PI positive cells from WT and UCP1KO primary brown adipocytes, following a 16hr fasting at 5mM glucose then 6hr culture with 25mM 2-DG or 5mM glucose, at day 8 of differentiation. About 500 cells from each group are counted. **e** WT and UCP1KO primary brown adipocytes were differentiated for 8 days and the amount of LDH activity in the medium following a 16hr fasting at 5mM glucose then 6hr culture with 25mM 2-DG or 5mM glucose was determined. n = 4 biologically independent samples. **f** The mitochondria from wild type male BAT were crosslinked with DSP or without, then lysed and immunoprecipitated with STX4 antibody, and then blotted with STX4, SNAP23, UCP1, TOM70 and COX IV antibody. **g** Immunoblot of STX4, UCP1, ATP5A and Vinculin brown adipose tissue from 4-month-old wildtype male mice housing under room temperature (WT-RT) or thermoneutrality (29℃, WT-NT) for 1 month. **h** Quantitation of UCP1 and STX4 protein in brown adipose tissue. n = 4 mice. **i** Immunoblot of STX4, UCP1 and COX IV in isolated mitochondria from wildtype brown adipose tissue maintained at either room temperature (WT-RT) or thermoneutrality (WT-TN) for 1 month. The mitochondria were treated with 20μg/μl proteinase K for 20 min. **j** Quantitation of UCP1 and STX4 protein levels in the mitochondria WT brown adipose tissue. n = 4 mice. All data represent the mean ± SEM. **P* < 0.05, ***P* < 0.01 and ****P* < 0.001, by two-tailed student's *t* test (b, c, e, h, j) or two-way ANOVA (d).
