# Supplementary material for: Involution of brown adipose tissue through a Syntaxin 4 dependent pyroptosis pathway
Source: Nat Commun. 2024 Apr 2;15:2856. doi: 10.1038/s41467-024-46944-y (PMC10987578; doi:10.1038/s41467-024-46944-y)
Supplement: Supplementary file 3 — Reporting Summary [file 41467_2024_46944_MOESM3_ESM.pdf]

nature portfolio

Corresponding author(s): Daorong Feng, NCOMMS-23-26676B
Last updated by author(s): Jan 31, 2024

Reporting Summary

Nature Portfolio wishes to improve the reproducibility of the work that we publish. This form provides structure for consistency and transparency in reporting. For further information on Nature Portfolio policies, see our Editorial Policies and the Editorial Policy Checklist.

Statistics

For all statistical analyses, confirm that the following items are present in the figure legend, table legend, main text, or Methods section.

n/a	Confirmed	
☐	☒	The exact sample size (*n*) for each experimental group/condition, given as a discrete number and unit of measurement
☐	☒	A statement on whether measurements were taken from distinct samples or whether the same sample was measured repeatedly
☐	☒	The statistical test(s) used AND whether they are one- or two-sided<br>*Only common tests should be described solely by name; describe more complex techniques in the Methods section.*
☐	☒	A description of all covariates tested
☐	☒	A description of any assumptions or corrections, such as tests of normality and adjustment for multiple comparisons
☐	☒	A full description of the statistical parameters including central tendency (e.g. means) or other basic estimates (e.g. regression coefficient) AND variation (e.g. standard deviation) or associated estimates of uncertainty (e.g. confidence intervals)
☐	☒	For null hypothesis testing, the test statistic (e.g. *F*, *t*, *r*) with confidence intervals, effect sizes, degrees of freedom and *P* value noted<br>*Give P values as exact values whenever suitable.*
☒	☐	For Bayesian analysis, information on the choice of priors and Markov chain Monte Carlo settings
☒	☐	For hierarchical and complex designs, identification of the appropriate level for tests and full reporting of outcomes
☒	☐	Estimates of effect sizes (e.g. Cohen's *d*, Pearson's *r*), indicating how they were calculated

Our web collection on statistics for biologists contains articles on many of the points above.

Software and code

Policy information about availability of computer code

Data collection

The following tools were used for data collection:
-TH-8 Thermometer (Physitemp Instruments)
-JEOL 1400Plus Transmission Electron Microscope (JEOL Ltd.)
-XF24 extracellular flux analyzer (Seahorse Biosciences)
-Spectrophotometer (Nanodrop 2000c, Thermo Fisher)
-QuantStudioTM 6 Flex Real-Time PCR System (Thermo Fisher Scientific)
-iBlot 2 W estern Blot Transfer system (Leica, NY)
-QTRAP 6500 LC-MS/MS System (Sciex)
-Leica SP8 Laser Confocal Microscope (Leica)
-Glucose Meter (Infinity, US Diagnostics)
-Microplate reader (lnfinite-M200)
-MoFloxXDP Cell Sorter (Beckman Coulter, Inc.)

Data analysis

The following software were used for data analysis:
-Image J software (version 2.0.0) was used for quantitative analysis.
-Cell Ranger Software (6.1.2) and Loupe Browser (6.4.1) were used for analysis of single nuclei data.
-Graph Pad Prism (version 8.1) were used for statistical analysis.
-R (version 4.0.4), R packages DESeq2 and clusterProfiler (version 4.8.2) were used for gene set enrichment analysis.
All the replicate experiments (including cell and mouse-based experiments) were biological replicates, which were repeated at least three times. Data are presented as means ± SEM and individual data points are plotted. The differences between two groups were evaluated using

unpaired two-tailed Student's t tests, and multiple group comparisons were conducted by one -way or two-way ANOVA followed by Tukey's multiple comparisons test. The statistical analyses were made at significance levels as follows: ns, not statistically significant; *p <0.05; **p <0.01; ***p <0.001, and ****p <0.0001. Statistical analyses were performed in EXcel or GraphPad PRISM 8.1. The images were created by Adobe Illustrator 2021 software.

For manuscripts utilizing custom algorithms or software that are central to the research but not yet described in published literature, software must be made available to editors and reviewers. We strongly encourage code deposition in a community repository (e.g. GitHub). See the Nature Portfolio guidelines for submitting code & software for further information.

Data

Policy information about availability of data

All manuscripts must include a data availability statement. This statement should provide the following information, where applicable:

- Accession codes, unique identifiers, or web links for publicly available datasets
- A description of any restrictions on data availability
- For clinical datasets or third party data, please ensure that the statement adheres to our policy

The authors declare that the data supporting the findings of this study are available within the manuscript, its supplementary information and the Source Data file. All data are publicly accessible. The Source data file has been provided.

Research involving human participants, their data, or biological material

Policy information about studies with human participants or human data. See also policy information about sex, gender (identity/presentation), and sexual orientation and race, ethnicity and racism.

Reporting on sex and gender	no human participants were involved.
Reporting on race, ethnicity, or other socially relevant groupings	no human participants were involved.
Population characteristics	no human participants were involved.
Recruitment	no human participants were involved.
Ethics oversight	no human participants were involved.

Note that full information on the approval of the study protocol must also be provided in the manuscript.

Field-specific reporting

Please select the one below that is the best fit for your research. If you are not sure, read the appropriate sections before making your selection.

☒ Life sciences ☐ Behavioural & social sciences ☐ Ecological, evolutionary & environmental sciences

For a reference copy of the document with all sections, see nature.com/documents/nr-reporting-summary-flat.pdf

Life sciences study design

All studies must disclose on these points even when the disclosure is negative.

Sample size	The n number for all experiments, including animal experiments and in vitro experiments were listed in the figure legends. The sample size was chosen based on our prior studies showed sufficient statistical power for in vitro experiments and animal experiments.
Data exclusions	No samples or animals were excluded from analyses.
Replication	All animal experiments were repeated at least twice and in vitro experiments were repeated at least three times. All results are reproducible and representative data were showed in the figures or supplementary files.
Randomization	Animals were allocated to their respective group after wean by a blinded investigator. For other experiments, including cell experiments, before performing the corresponding treatment, samples were randomly assigned to control and experimental groups by an investigator blinded to subsequent experimental information. The standard laboratory procedures were strictly followed to keeping the experimental environment and facilities consistent and performed under the same conditions.
Blinding	Investigators were blinded to group allocation during data collection, image quantification and data analysis.

Reporting for specific materials, systems and methods

We require information from authors about some types of materials, experimental systems and methods used in many studies. Here, indicate whether each material, system or method listed is relevant to your study. If you are not sure if a list item applies to your research, read the appropriate section before selecting a response.

Materials & experimental systems

n/a	Involved in the study
☐	☒ Antibodies
☐	☒ Eukaryotic cell lines
☒	☐ Palaeontology and archaeology
☐	☒ Animals and other organisms
☒	☐ Clinical data
☒	☐ Dual use research of concern
☒	☐ Plants

Methods

n/a	Involved in the study
☒	☐ ChIP-seq
☒	☐ Flow cytometry
☒	☐ MRI-based neuroimaging

Antibodies

Antibodies used

Vinculin (Abcam,#ab18508), Caspase 1 (AdipGen Life Sciences, #AG-20B-0042); Caspase 1(ThermoFisher, #PA5-38100; AdipGen Life Sciences, #ag-20b-0044); Caspase 3 (Cell Signaling, #9661); Caspase 11 (Abcam, #ab180673); Syntaxin 4 (Synaptic Systems, #110042); Tubulin (Cell signaling, #2144); Hmgb1 (Abcam, #ab67281); UCP1 (Abcam, #ab10983); Nlrp1b (Novus Biologicals, #NBP1-54899); Cleaved Nlrp1b (AdipGen Life Sciences, #AG-20B-0084); PGAM1 (Cell signaling, # 12098); HSP70 (Abcam, #abab181606); AIM2 (Abcam, #ab93015); NLRP3 (AdipGen Life Sciences, #AG-20B-0006); GAPDH (Abcam, #ab8245); GM130 (Cell signaling, #12480); Caveolin (Cell signaling, # 3238); Myc (Cell signaling, # 2278); Flag (Sigma, #F1804); SNAP23 (Abcam, #ab3340); ATP5A (Abcam, #ab151229); TOM70 (Cell signaling, #65619); COXIV (Cell signaling, #4844); TOM20 (Cell signaling, #42406); OXPHOS (Abcam, #ab110413); Perilipin 1 (Cell signaling, #3470), F4/80 (Cell signaling, #70076)

Validation

The antibodies including Vinculin, Caspase 1, Caspase 3, Caspase 11, Syntaxin 4, Tubulin, UCP1, Nlrp1b, Cleaved Nlrp1b, PGAM1, AIM2, NLRP3, GAPDH, GM130, Caveolin, Myc, Flag, SNAP23, ATP5A, TOM70, COXIV, TOM20 and OXPHOS were validated for the western blotting of mouse samples on the websites of the associated companies (https://www.thermofisher.com/, https://www.cellsignal.com/, https://www.abcam.com/, https://www.sysy.com, https://adipogen.com,https://www.novusbio.com,). Ucp1 and F4/80 antibodies were validated for the immunohistochemical staining of mouse samples on the websites of the companies (https://www.cellsignal.com/,https://www.abcam.com/). The antibodies including Ucp1, Perilipin 1 and Hmgb1 were validated for the immunofluorescence staining of mouse samples on the websites of the associated companies (https://www.thermofisher.com/, https://www.cellsignal.com/, https://www.abcam.com/).

Eukaryotic cell lines

Policy information about cell lines and Sex and Gender in Research

Cell line source(s)

Stx4fl/fl cell line, we developed from brown adipose tissue of 4-day-old mouse Stx4fl/fl pups.

Authentication

The cells were then immortalized with SV40 lentivirus and transfected with tamoxifen inducible Cre retrovirus. Single clones were selected and assessed for their ability to differentiate. Only clones with good differentiation were retained for further experiments.

Mycoplasma contamination

negative for mycoplasma contamination.

Commonly misidentified lines
(See ICLAC register)

No misidentified lines were used in the study.

Animals and other research organisms

Policy information about studies involving animals; ARRIVE guidelines recommended for reporting animal research, and Sex and Gender in Research

Laboratory animals

The C57BL/6J mice used in this study were obtained from the Jackson Laboratory (000664, JAX). The Stx4fl/fl mice were generously provided by Dr. Adachi Roberto, while the Ucp1-Cre mice were obtained from the Jackson Laboratory (024670, JAX). The Ucp1-cre/ERT2 mice were gifted by Dr. Christian Wolfrum.
Rosa26-Stx4a KI mice: we developed this mice by inserting a cassette consisting of Ad-SA (a splicing acceptor)-loxp-tPA (triple polyA signal)-loxp-mouse Stx4a CDS-PolyA signal in sequence, we used the CRISPR/HDR strategy to target intron 1 of the Rosa26 locus. Microinjections to obtain zygotes and live mouse pups were performed by Albert Einstein Gene Modification Facility and Transgenic Mouse Facility.
The age of animals is from 2 month to 24 months and described in the manuscript.

Wild animals

The study did not involve any wild animal.

Reporting on sex

The experiments in the study were done in both sexes. Relevant information is described detailly in the manuscript.

Field-collected samples

No field-collected samples were used in the study.

Ethics oversight	All animal studies were conducted in accordance with the guidelines of the Institutional Animal Care and Use Committee at Albert Einstein College of Medicine (our approved protocol number: 00001103).

Note that full information on the approval of the study protocol must also be provided in the manuscript.

Plants

Seed stocks	no plants were involved.
Novel plant genotypes	no plants were involved.
Authentication	no plants were involved.
